# Prevalence of depressive tendencies among college students and the influence of attributional styles on depressive tendencies in the post-pandemic era

**DOI:** 10.3389/fpubh.2024.1326582

**Published:** 2024-01-25

**Authors:** Ming-ming Luo, Ming Hao, Xu-huan Li, Jing Liao, Chun-mei Wu, Qi Wang

**Affiliations:** ^1^Jiangxi Cancer Hospital, The Second Affiliated Hospital of Nanchang Medical College, Jiangxi Cancer Institute, Nanchang, China; ^2^Department of Health Statistics, School of Public Health and Health Management, Gannan Medical University, Ganzhou, China; ^3^The 4th Affiliated Hospital, Jiangxi Medical College, Nanchang University, Nanchang, China

**Keywords:** attribution, depressive tendency, depression, academic, medic

## Abstract

**Introduction:**

Depression symptoms are prevalent globally, including China, with a notable impact on college students. This study aims to not only estimate the prevalence of depressive tendencies and attributional styles among college students in the post-pandemic era but also explore the relationship between the two factors. The findings of this study can provide new insights into early intervention and support services for individuals exhibiting tendencies toward depression.

**Methods:**

The survey was administered to college students from various academic backgrounds at a specific university in southern Jiangxi Province by employing two scales to investigate depressive tendencies and attributional styles. Depressive tendency was evaluated using the Patient Health Questionnaire-9 (PHQ-9), while the attributional styles were assessed using the Multidimensional-Multiattributional Causality Scale (MMCS). Univariate analysis and multiple logistic regressions were conducted to identify the related factors of depressive tendency.

**Results:**

A relatively high (48.9%) prevalence of depression among college students was found in this study. College students with higher grades (OR = 1.574, 95%CI: 1.369–1.810), profession of medicine and allied health sciences (OR = 1.779, 95%CI: 1.203–2.629), experiencing higher study stress (OR = 2.006, 95%CI: 1.601–2.514), and having poor physical condition (OR = 1.527, 95%CI: 1.247–1.869) were identified as risk factors for depressive tendency. The correlation between higher grades and increased learning pressure, coupled with poorer physical condition, heightens the vulnerability of college students to depression. Moreover, the more they attribute these experiences to achievement effort (OR = 0.897, 95%CI: 0.828–0.972), achievement ability (OR = 0.903, 95%*CI*: 0.838–0.972), and affiliation context (OR = 0.919, 95%CI: 0.860–0.982), the less likely they are to develop depression.

**Conclusion:**

In the group of college students, especially those in higher grades, profession of medicine and allied health sciences or experiencing high learning pressure and poor physical condition, emphasizing the significance of their mental well-being becomes crucial. Offering suitable support and assistance is essential. Additionally, fostering the cultivation of positive attributional and coping strategies by attributing difficulties to controllable factors and instilling a belief in their ability to overcome challenges can help reduce the risk of depression.

## 1 Introduction

Depression, also known as depressive disorder, is characterized by persistent psychological experiences of loss, sadness, and hopelessness in an individual’s life. It is a prevalent emotional disorder that is commonly encountered (1, 2). At present, the main diagnostic systems for depression include Diagnostic and Statistical Manual of Mental Disorders, 5th Edition (DSM-5) (3) and International Classification of Diseases, 11th edition (ICD-11) (4). The World Health Organization’s 2017 report on “Depression and Other Common Mental Disorders” revealed a substantial growth rate of 18% in the number of individuals diagnosed with depression between 2005 and 2015 (5). By 2023, it is estimated that approximately 280 million individuals globally will be contending with the heavy burden of depression, and this figure is expected to continue rising in the foreseeable future due to the impact of the coronavirus disease 2019 (COVID-19) pandemic and the rapid evolution of society (6–9). Consequently, depression has emerged as a leading contributor to the global burden of mental illness in recent years (6). It is noteworthy that depression typically evolves through a prolonged developmental process rather than a sudden onset (10). Influenced by various factors such as physiology, psychology, and society, individuals may exhibit depressive tendencies such as feelings of loss and self-denial. If these symptoms persist and significantly impact personal life and daily functioning, there is a potential for the condition to further progress into depression.

In China, the lifetime prevalence rate of depression is 6.9%, and the 12-month prevalence rate is 3.6% (11). Based on these data, it is estimated that there would be over 90 million patients suffering from depression in China by 2019 (11). Research has shown that 72% of depressed patients in China are diagnosed before the age of 25, with most being diagnosed in middle school or university (12). Compared to primary and secondary school students, college students face various psychological pressures and challenges, including academic and employment pressure, interpersonal problems, and life changes, which can significantly impact their mental health (13). Studies also suggest that during college, approximately 10–30% of students experience depressive tendencies, with some even developing depressive disorders (14). Upon further analysis of research data 1 year after the outbreak of the COVID-19 pandemic, it has been discovered that there is a more pronounced shift in the prevalence of severe depression and anxiety disorders among young individuals (particularly university students) relative to their older adult counterparts (15). This can be primarily attributed to factors such as school closures and social restrictions, which have impeded collective learning and peer interaction for the younger generation, thereby consequently affecting their academic performance and social aptitude (16). Additionally, during this period, the youth are relatively more susceptible to the risk of unemployment when compared to the older adult population (17). Depression can significantly impact an individual’s physical, psychological, and social well-being, leading to diminished academic and work performance, interpersonal challenges, physical health complications, and an increased risk of suicide (18–20). Therefore, the prevalence of depression among college students has become a worldwide concern, particularly in the post-pandemic era, and addressing this issue is critical for the well-being of individuals and society.

Numerous studies have shown that utilizing negative attribution can contribute to depressive moods, thereby impacting the mental health of individuals (21–23). Attributional style, sometimes known as explanatory style, refers to the way people explain the reasons for events that occur in their lives (24). For instance, when individuals attribute their failure, such as performing poorly on a final exam, to their own perceived lack of ability, it can foster a negative belief system that undermines their efforts. Addressing this negative attributional style is a key aspect of interventions aimed at tackling depression among college students, with a primary emphasis on behavioral and cognitive interventions. However, the association between attributional style and depression among college students remains inconclusive from a cognitive psychology standpoint. Therefore, it is imperative to identify predispositions toward depression in university students and explore the associated influencing factors. This enables the implementation of targeted early intervention measures and depression management strategies. Consequently, this study aims to examine the prevalence of depressive tendencies and attributional styles among college students while investigating the impact of attributional style on depressive tendencies. By drawing upon psychological insights, this research seeks to offer innovative perspectives for early intervention and support services related to depressive tendencies.

## 2 Methods

### 2.1 Study population

In the present study, students from various grades at a university located in southern Jiangxi Province were selected as research subjects. Prior to the investigation, the research objectives and content were introduced. The population selection criteria for this study comprised university students aged 18–25 from diverse academic disciplines. Participants who were unwilling to cooperate, or had severe mental health issues and/or poor physical health such that they were incapable of cooperating with the investigations were excluded. A total of 719 students were selected as research participants through stratified cluster random sampling, and informed consent was obtained from all participants, thus ensuring their voluntary participation in the study.

### 2.2 Data collection and procedures

The survey was conducted from January to April 2023, when the COVID-19 epidemic had entered the post-pandemic stage. The survey mainly consisted of two primary stages: data collection using the paper-and-pencil interview method and quality control, which included a design and pilot study, investigator training, on-site investigation, as well as verification of data integrity and authenticity. Depressive symptoms were assessed with the Patient Health Questionnaire-9 (PHQ-9), while the attributional styles of college students were appraised using the Multidimensional-Multiattributional Causality Scale (MMCS). The demographic information of participants was also collected through a general questionnaire.

The PHQ-9 is a widely utilized scale designed to evaluate the presence and severity of depressive symptoms (25). It encompasses dimensions such as emotional well-being, sleep patterns, concentration levels, and suicidal ideation. Comprising nine items, the scale adopts a four-level response format (none, a few days, over a week, and almost every day), with corresponding scores of 0, 1, 2, and 3, respectively. Based on the cumulative scores, depressive tendencies are categorized as follows: 0–4 points indicate the absence of depression; 5–9 points indicate mild depression; 10–14 points indicate moderate depression; 15–19 points indicate moderately severe depression; and 20–27 points indicate severe depression. The PHQ-9 has been extensively validated by scholars, demonstrating strong reliability and validity (26–28). In the present survey, the Cronbach’s alpha coefficient and the Guttman split-half coefficient for the PHQ-9 was 0.872 and 0.827, respectively, indicating high internal consistency. Furthermore, the Kaiser–Meyer–Olkin (KMO) coefficient of the PHQ-9 was 0.915, and Bartlett’s test was statistically significant (*p* < 0.001), indicating a good validity of the scale.

The MMCS is a psychological assessment tool proposed by psychologist Lefcourt in 1979 (29). It helps researchers gain insights into individuals’ perceptions of causal attributions for events through a multidimensional and multifactorial approach. This, in turn, aids in understanding the psychological processes and cognitive patterns underlying individuals’ perspectives on event occurrences. The MMCS is a 48-item scale consisting of two components: achievement (24 items) and affiliation (24 items). Each component is divided into four dimensions, namely, “attributed to ability,” “attributed to effort,” “attributed to luck,” and “attributed to situation.” Within each dimension, three items reflect positive attribution for successful outcomes, while three other items reflect negative attribution for unsuccessful outcomes (see Table 1 for specific details). Each item is rated on a 5-point Likert scale, with responses ranging from “strongly agree” (4 points) to “completely disagree” (0 points). Higher scores indicate a stronger tendency toward attribution within a particular dimension. The MMCS has shown strong reliability and validity in various studies (30). In this study, the Cronbach’s Alpha coefficient and the Guttman split-half coefficient of the MMCS were 0.940 and 0.883, respectively. The KMO coefficient was 0.942 and Bartlett’s test was statistically significant (*p* < 0.001), hence suggesting that this scale had good reliability and validity.

**Table 1:** Items and dimensions of the MMCS scale.

Dimension	Achievement attribution		Affiliation attribution	
	Positive result items	Negative result items	Positive result items	Negative result items
Ability	11, 27, 43	3, 19, 35	15, 31, 47	7, 23, 39
Effort	9, 25, 41	1, 17, 33	13, 29, 45	5, 21, 37
Context	6, 22, 38	14, 30, 46	2, 18, 34	10, 26, 42
Luck	8, 24, 40	16, 32, 48	4, 20, 36	12, 28, 44

### 2.3 Sample size

In accordance with the principle established by Kendall, it is recommended that sample sizes should encompass 5–10 times the number of variables (31). This study utilized 2 scales consisting of 57 items. To account for potential rejection rates and incomplete questionnaires, we increased the sample size by 15%. Consequently, the final minimum sample size required is 656 people.

### 2.4 Statistical analysis

Quantitative data were described using mean ± standard deviations (Mean ± SD), and intergroup comparisons were conducted using *t*-tests. Qualitative and hierarchical data were presented as N (%), and differences were evaluated using the chi-square test or Wilcoxon rank-sum test. The composition of depressive tendencies in the population and the scores of each attributed dimension were depicted using pie charts and radar charts, respectively. Subsequently, risk factors for depression were identified using a binary logistic regression analysis with the variables that were statistically significant in the univariate analysis. All statistical analyses were conducted at a significance level of 0.05, with *p* < 0.05 indicating statistical significance.

## 3 Results

### 3.1 The distribution of depressive tendencies among the study subjects

**Figure 1:**
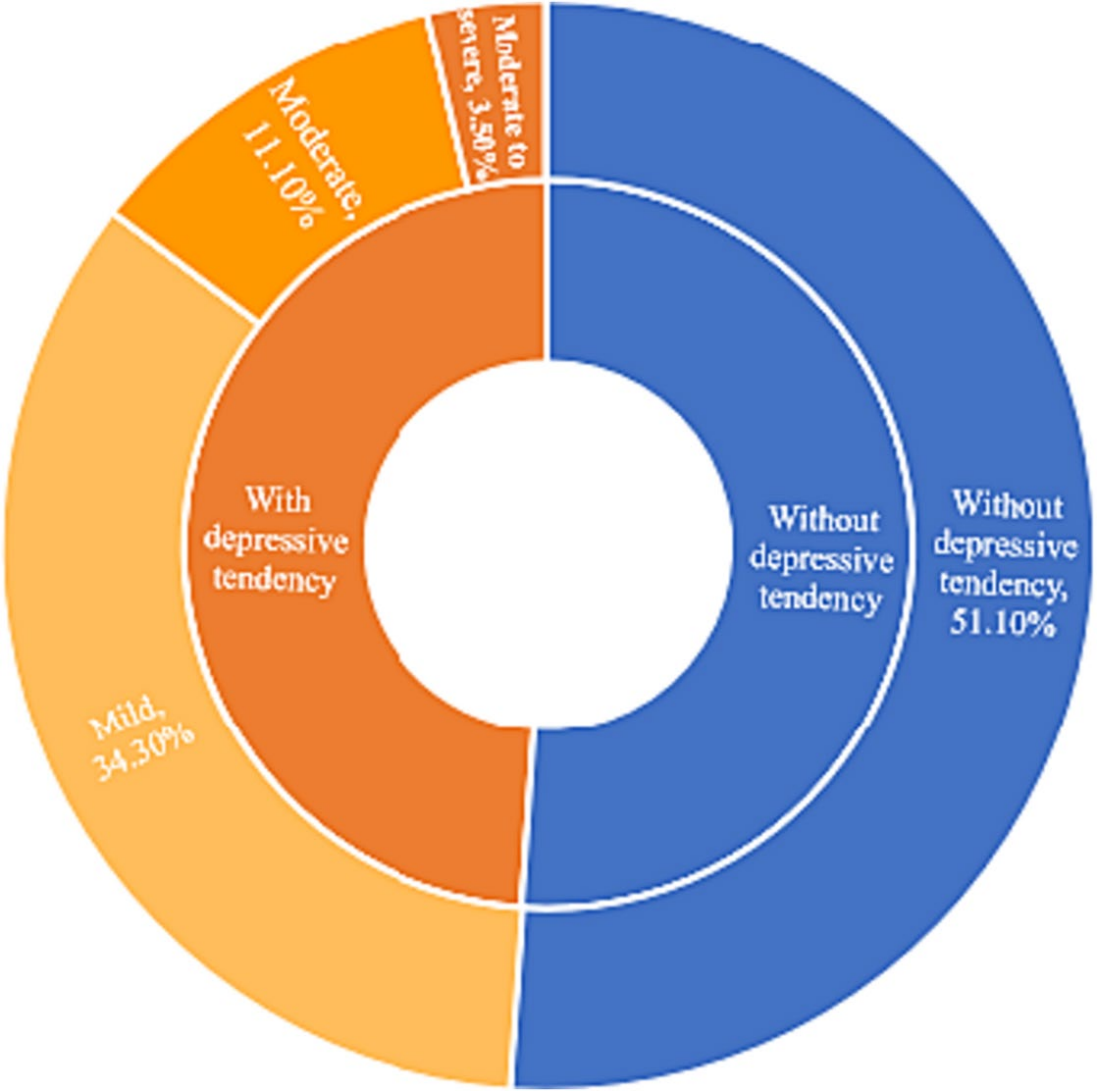
Composition chart of depression tendency of college students.

In this study, a total of 719 questionnaires were sent out, and 698 questionnaires were successfully recovered, resulting in a response rate of 97.08%. Out of the returned questionnaires, 679 questionnaires were considered valid, with an effective rate of 94.44%. Based on the criteria of the PHQ-9, a total of 332 participants were found to have depressive tendencies, accounting for 48.9% of the total survey subjects. Among them, 233 individuals (34.3% of the total surveyed) exhibited mild depressive tendencies, while 24 individuals (14.6% of the total surveyed) were found to have moderate-to-severe depressive tendencies. No participants were identified as having severe depressive tendencies (see Figure 1). Notably, there were significant differences in depressive tendencies observed among students of different grades, profession, physical conditions, and levels of academic stress (*p* < 0.05). In terms of profession, we have divided it into two categories: medicine and allied health sciences, and non-medicine and allied health sciences. Medicine and allied health sciences profession were disciplines related to healthcare, such as medicine, nursing, pharmacy, and allied health professions, while non-medicine and allied health sciences profession refer to disciplines that do not involve healthcare. A comprehensive overview of the participants’ demographic characteristics and the distribution of depressive tendencies can be found in Table 2.

**Table 2:** General information and distribution of depression tendency of the participants.

General demographic characteristics	Category	Frequency	Depressive tendencies		*Z/χ* ^2^	*p*
			With	Without		
Gender	Male	332	154 (46.39)	178 (53.61)	1.638	0.201
	Female	347	178 (51.30)	169 (48.70)		
Grade	Freshman year	181	56 (30.94)	125 (69.06)	−8.634	<0.001
	Sophomore year	165	70 (42.42)	95 (57.58)		
	Junior year	152	65 (42.76)	87 (57.24)		
	Senior year	89	66 (74.16)	23 (25.84)		
	Last year	92	75 (81.52)	17 (18.48)		
Profession	Medicine and allied health sciences	499	263 (52.71)	236 (47.29)	10.935	0.001
	Non-medicine or allied health sciences	180	69 (38.33)	111 (61.67)		
Family economic situation	High-income	33	18 (54.55)	15 (45.45)	−0.738	0.461
	Middle to high income	76	43 (56.58)	33 (43.42)		
	Middle income	278	119 (42.81)	159 (57.19)		
	Low to middle income	208	104 (50.00)	104 (50.00)		
	Low-income	84	48 (57.14)	36 (42.86)		
Physical condition	Very good	103	48 (46.60)	55 (53.40)	−5.028	<0.001
	Preferably	262	97 (37.02)	165 (62.98)		
	Commonly	236	127 (53.81)	109 (46.19)		
	Poor	69	54 (78.26)	15 (21.74)		
	Very poor	9	6 (66.67)	3 (33.33)		
Learning pressure	Very high	65	52 (80.00)	13 (20.00)	−8.527	<0.001
	High	207	139 (67.15)	68 (32.85)		
	Commonly	330	112 (33.94)	218 (66.06)		
	Low	62	25 (40.32)	37 (59.68)		
	Very low	15	4 (26.67)	11 (73.33)		

### 3.2 Scores of attribution in various dimensions of the study participants

**Figure 2:**
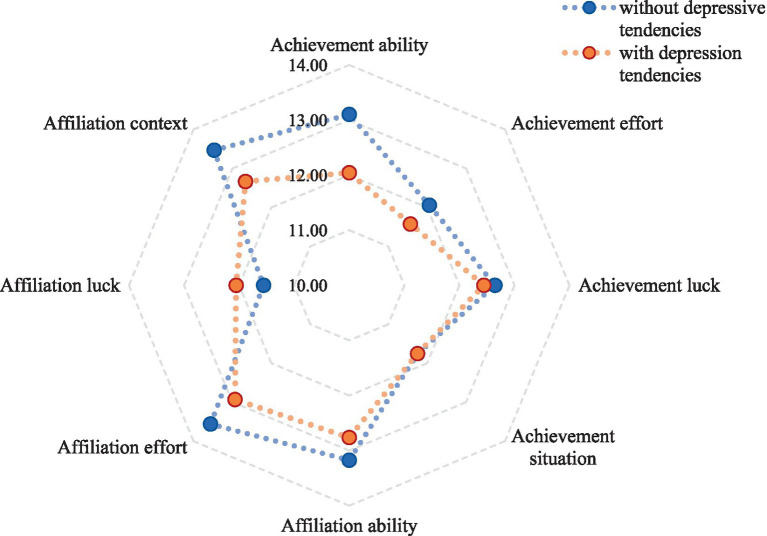
Radar chart of scores on 8 dimensions of attribution styles among participants with different depressive tendency.

The scores of each dimension in the MMCS of study participants were shown in Table 3. In students with different depressive tendencies, there were significant differences in the scores across all dimensions except for achievement luck and achievement context (*p* < 0.05), as shown in Figure 2.

**Table 3:** Scores of attribution in various dimensions of the participants.

		Depressive tendencies		*t*	*p*
		Without	Without		
Achievement	Ability	12.04 ± 2.39	13.10 ± 2.46	5.691	<0.001
	Effort	11.57 ± 2.23	12.06 ± 2.35	2.759	0.006
	Luck	12.45 ± 2.48	12.65 ± 2.42	1.062	0.289
	Context	11.75 ± 2.24	11.76 ± 2.44	0.011	0.991
Affiliation	Ability	12.77 ± 2.19	13.18 ± 2.47	2.287	0.022
	Effort	12.93 ± 2.46	13.56 ± 2.52	3.290	0.001
	Luck	12.05 ± 2.20	11.56 ± 2.29	−2.888	0.004
	Context	12.67 ± 2.53	13.47 ± 2.87	3.873	<0.001

### 3.3 Analysis on the influencing factors of depressive tendency

To investigate the influencing factors of depressive tendencies, this study employed binary logistic regression analysis with depressive tendencies as the dependent variable and demographic characteristics, as well as indicators that exhibited statistically significant differences in attributive dimensions, as independent variables. The specific assignment table can be found in Table 4. The findings of the logistic regression analysis revealed that several factors significantly affect depressive tendency in college students, including grade, physical condition, profession, academic pressure, achievement ability, achievement effort, and affiliation context. College students with higher grades (OR = 1.574, 95%CI: 1.369–1.810), profession of medicine and allied health sciences (OR = 1.779, 95%CI: 1.203–2.629), experiencing higher study stress (OR = 2.006, 95%CI: 1.601–2.514), and having poor physical condition (OR = 1.527, 95%CI: 1.247–1.869) were identified as risk factors for depression. Conversely, higher scores in attributing to achievement effort (OR = 0.897, 95%CI: 0.828–0.972), achievement ability (OR = 0.903, 95%CI: 0.838–0.972), and affiliation context (OR = 0.919, 95%CI: 0.860–0.982) served as protective factors against depressive tendencies among college students (all the *p* < 0.05) (see Table 5).

**Table 4:** Assignment of variable for logistic regression analysis.

Variable	Variable assignment
Grade	Freshman year =1, Sophomore year =2, Junior year =3, Senior year =4, Last year = 5
Profession	medicine and allied health sciences =1, non-medicine or allied health sciences =2
Physical condition	Very good =1, Preferably =2, Commonly = 3, poor =4, very poor =5.
Academic pressure	Very high =1, High =2, Commonly =3, Low =4, Very low =5
Achievement ability	Continuous variable
Achievement effort	Continuous variable
Affiliation ability	Continuous variable
Affiliation effort	Continuous variable
Affiliation luck	Continuous variable
Affiliation context	Continuous variable

**Table 5:** Binary logistic regression analysis of factors affecting depressive tendency.

	*b*	*S_b_*	Wald *χ*^2^	*p*	OR	OR 95% CI	
						Lower	Upper
Constant	−1.909	1.060	3.246	0.072	0.148		
Grade	0.454	0.071	40.634	<0.001	1.574	1.369	1.810
Physical condition	0.423	0.103	16.830	<0.001	1.527	1.247	1.869
Profession	0.576	0.199	8.333	0.004	1.779	1.203	2.629
Academic stress	0.696	0.115	36.599	<0.001	2.006	1.601	2.514
Achievement effort	−0.108	0.041	7.009	0.008	0.897	0.828	0.972
Achievement ability	−0.103	0.038	7.274	0.007	0.903	0.838	0.972
Affiliation context	−0.085	0.034	6.318	0.012	0.919	0.860	0.982

## 4 Discussion

The present study reveals that a significant proportion (48.9%) of college students are susceptible to depression in the post-pandemic era. Students with higher academic years, higher academic stress, and poorer physical condition are more likely to exhibit depressive tendencies. Conversely, students who attribute events to their own achievement efforts, abilities, and social affiliations are less prone to experiencing such tendencies.

The university stage is widely acknowledged as a critical transitional period characterized by numerous challenges and pressures, including academic pressure, interpersonal problems, and employment concerns (13, 32). In such a context, college students are prone to negative emotions, especially depressive tendencies. If not intervened, the depressive tendencies of college students can easily develop into depression, which not only affects personal physical and mental health, as well as academic and interpersonal relationships, but also brings huge economic burden to society. The widespread impact of the COVID-19 pandemic may have further exacerbated the psychological challenges for college students, including academic stress, social isolation, and financial strains, thereby influencing their vulnerability to depression (33). Therefore, understanding the depressive tendency level of college students and exploring the corresponding influencing factors can better focus on the mental health of college students and provide necessary support and assistance. Based on the survey conducted for this study, it was found that college students exhibited a relatively high propensity toward depression, with a prevalence rate of 48.9%. These findings align with the results reported by Yang et al. (34). Although mild depressive tendencies are predominant and no severe depressive tendencies are detected, it is important to note that the proportion of college students with depressive tendencies approaching 50% suggests a relatively high prevalence. To prevent the escalation of depressive tendencies among college students, adopting targeted measures for early intervention is imperative. Therefore, it might be essential to determine the influencing factors on depression tendency.

This study found no gender differences in the tendency toward depression among college students, contrasting with numerous studies that have shown higher levels of depression among females compared to males (35, 36). This discrepancy may be attributed to the changing social attitudes toward mental health, leading to increased awareness and willingness among male college students to seek help. Consequently, the detection of depressive tendencies between genders may gradually become more balanced. Furthermore, the findings indicate that depression rates vary across different academic grades, with higher grades exhibiting a significantly higher proportion of depressive tendencies, consistent with prior research (37). This can be because senior students face more academic and future pressures such as graduation and employment, leading to feelings of anxiety and unease. These findings align with the conclusions of other researchers (38, 39). In our study, we also find that students pursuing a profession in medicine and allied health sciences are more prone to depression, perhaps because they often require more study time and energy investment, and the monotonous learning stages and heavy academic pressures are more likely to engender depressive tendencies. There are many sources of pressure during the study stage, and when college students are unable to cope with high-intensity academic pressure, they are also prone to negative emotions. The long-term accumulation of negative emotions can easily lead to depressive tendencies among students (40). Therefore, in the process of learning, educational institutions should provide students with support and assistance in managing academic pressure, employment, and other aspects, such as psychological counseling, social support, and so on. Furthermore, those with poorer physical conditions are also more likely to experience depressive tendencies (41, 42). This is because as physical condition worsens and discomfort increases, college students reduce their interaction and communication with others, leading to a decrease in self-worth. This not only exacerbates feelings of sadness and anxiety but also contributes to a sense of frustration and low self-esteem, thus increasing the risk of depression. It should be noted that there is a mutual influence and vicious cycle between poor physical condition and depression: those with poor physical health are more prone to depressive tendencies, and conversely, depression itself may further worsen physical health. Therefore, for students with poorer physical conditions, timely support and assistance should be provided to promote physical treatment and rehabilitation, while also emphasizing the management and treatment of mental health.

Besides academic pressure and physical condition, college students’ attribution of events also plays a significant role in their susceptibility to depression. Attribution refers to the way individuals speculate and explain the causes of events or situations (43). To gain insights into college students’ attributional styles and their impact on depressive tendencies, educational institutions can implement strategies such as psychological education and cognitive training. These interventions aim to promote healthy attributional styles, enhance students’ psychological well-being, and prevent the occurrence of depression. In this study, except for academic luck and academic situational attribution, there were differences in the scores of other attributional styles between individuals with or without depressive tendencies. Logistic regression analysis revealed that attributing events to achievement ability, achievement effort, and affiliation context was associated with a lower likelihood of experiencing depression. In the MMCS scale, the dimensions of both ability and effort attributions belong to internal attribution, which refers to attributing events to internal factors. However, luck and context attributions belong to external attribution, which refers to attributing events to external factors, specifically the influence of the external environment (29). Achievement ability and effort are considered positive internal academic attributions. Students who attribute difficulties and setbacks to achievement ability and effort believe that they can cope with and overcome difficulties through effort and ability. This also means that students are more inclined to face problems positively and seek solutions. Affiliation context is a subset of external factors of interpersonal attribution. Attributing events to interpersonal situations means that students recognize the influence of their environment and interpersonal relationships on their emotions and psychological state. This recognition can prompt students to actively seek interpersonal support and help, thereby alleviating negative emotions, receiving emotional support and encouragement, and reducing the occurrence of depressive tendencies. Therefore, attributing difficulties and setbacks to achievement ability, achievement effort, and affiliation context helps students reflect on ways to enhance their academic capabilities, improve learning methods, or adjust interpersonal relationships to obtain better support. Guiding and encouraging students to develop this positive problem-solving attitude will help reduce the occurrence of depressive tendencies and assist individuals in better coping with stress and difficulties.

This study also has some limitations. First, this survey did not consider the impact of the COVID-19 pandemic when designing the survey questionnaire. However, for some senior students pursuing a profession in medicine and allied health sciences, they were directly involved in the treatment of COVID-19. Additionally, previous research has found an increased prevalence of negative psychological symptoms among healthcare workers (44, 45). Owing to the impact of the COVID-19 pandemic, various activities of college students, such as social interactions and physical exercise, have been restricted, which may also increase their tendency toward depression (46, 47). Our results also confirm that seniority, profession of medicine and allied health sciences, physical health status, and social interactions are associated with a higher tendency toward depression, which indirectly reflects the impact of the COVID-19 pandemic on depressive tendencies. Second, our research focused on college students within a specific geographic location, which might limit the generalizability of our findings to broader populations or different cultural contexts. It is essential to acknowledge the potential influence of cultural and regional factors on the prevalence of depressive tendencies and attributional styles (48). Third, the omission of mental health from the study may have an impact on the results, as it is likely associated with depressive tendencies. However, the assessment of mental health requires the use of scales, which further complicates the data collection process. Furthermore, given the personalized and sensitive nature of mental health information, it can lead to an increase in refusal rates. Additionally, the university conducts regular mental health assessments and interventions for students each year to ensure their well-being. Therefore, after careful consideration, we chose not to include mental health in the present study. Fourth, this study found that 3.5% of participants exhibited moderate to severe depressive tendencies, indicating an urgent need for intervention. However, the use of anonymous surveys in this study for a better understanding of participants’ true conditions prevented us from providing feedback, hallmarking another limitation of this study. In future research, we plan to optimize the survey process by assigning a specific identification number and QR code to each participant, thus enabling them to access their individual results by scanning the QR code and entering their personal identification number. This approach not only ensures result authenticity but also allows for the provision of personalized feedback.

In conclusion, the present study has identified several factors associated with depressive tendencies among college students. These findings emphasize the need for increased attention to mental health in specific subgroups, including seniors, students pursuing a profession in medicine and allied health sciences, as well as those facing high academic pressure or poor physical condition. It is crucial to provide appropriate support and assistance to these individuals. Additionally, fostering positive attribution methods and coping strategies that attribute difficulties to positive and controllable factors, as well as believing in one’s ability to overcome challenges, will help reduce the occurrence of depressive tendencies.

## Data availability statement

The raw data supporting the conclusions of this article will be made available by the authors, without undue reservation.

## Ethics statement

The studies involving humans were approved by Biomedical Research Ethics Committee of Gannan Medical University. The studies were conducted in accordance with the local legislation and institutional requirements. The participants provided their written informed consent to participate in this study. Written informed consent was obtained from the individual(s) for the publication of any potentially identifiable images or data included in this article.

## Author contributions

M-mL: Writing – original draft, Writing – review & editing, Methodology. MH: Conceptualization, Methodology, Supervision, Writing – review & editing. X-hL: Validation, Writing – review & editing. JL: Data curation, Investigation, Writing – review & editing. C-mW: Supervision, Validation, Writing – review & editing. QW: Conceptualization, Methodology, Project administration, Supervision, Writing – review & editing.
